# Ambigols Uncouple Oxidative Phosphorylation In Vitro

**DOI:** 10.1021/acschembio.5c00952

**Published:** 2026-04-02

**Authors:** Valerie I. C. Rebhahn, Clemens A. Wolf, Timo H. J. Niedermeyer

**Affiliations:** † Institute of Pharmacy, Pharmaceutical Biology, 9166 Freie Universität Berlin, Berlin 14195, Germany; ‡ Department of Biomedicine, University of Bergen, Jonas Lies Vei 91, Bergen 5020, Norway

## Abstract

Ambigols are natural products produced by the cyanobacterium *Symphyonema bifilamentata*. Especially ambigols A and C have a broad bioactivity spectrum. Therefore, ambigols have been discussed as promising compounds, e.g., for the development of new antibiotics. However, their mode of action is still unknown, although first steps have been undertaken toward its elucidation. Here, we show that ambigols A and C are uncouplers of oxidative phosphorylation. They dissipate the mitochondrial membrane potential and increase the oxygen consumption rate of HeLa cells. A further target is the membrane potential of Gram-negative bacteria. The disturbance of the electrochemical membrane potential in prokaryotic and eukaryotic cells shows the unspecific effects of typical uncouplers. These results provide a basis for a deeper understanding of the broad bioactivity spectrum of the ambigols, challenging the future development of ambigol-based compounds for clinical application.

## Graphical abstract

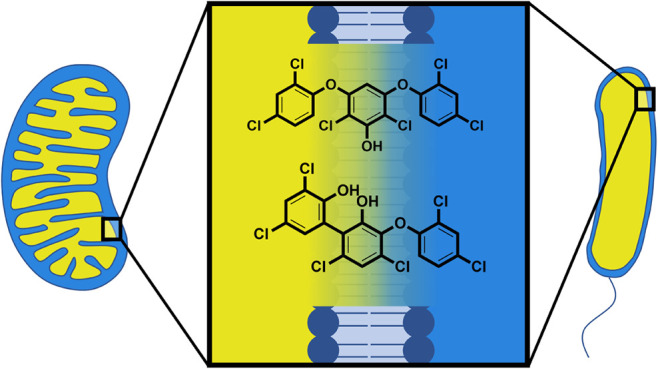

## Introduction

Uncouplers of oxidative phosphorylation disrupt the connection between the electron transfer chain and ADP phosphorylation.^1^ Several natural products of bacteria^2–4^ and plants^5–7^ have been found to be uncouplers, but little is known about uncouplers as specialized metabolites of cyanobacteria. The polybrominated diphenyl ethers (PBDEs) originating from the marine cyanobacterium *Hormoscilla spongeliae* were for long the only cyanobacteria-derived natural products with demonstrated uncoupling activity.^8–10^ Recently, we discovered that also aetokthonotoxin, produced by the cyanobacterium *Aetokthonos hydrillicola*, acts as an uncoupler and protonophore.^11^ Chemical structures of uncouplers feature strong electron-withdrawing groups, hydrophobic moieties, and dissociable groups, leading to characteristic lipophilic and weakly acidic physicochemical properties.^1,11^ These criteria are met by the already mentioned natural products. Also ambigols, produced by the cyanobacterium *Symphyonema bifilamentata* (formerly called *Fischerella ambigua* 108b)^12^ by radical coupling of 2,4-dichlorophenol building blocks,^13^ possess these physicochemical properties. As polychlorinated phenols linked via biaryl- and biaryl-ether-bonds (ambigol A, D, and E) or biaryl-ether-bonds (ambigol B and C), they are chemically closely related to the PBDEs and should have the required lipophilicity and weak acidity (Figure 1 and Table S1).

**Figure 1:**
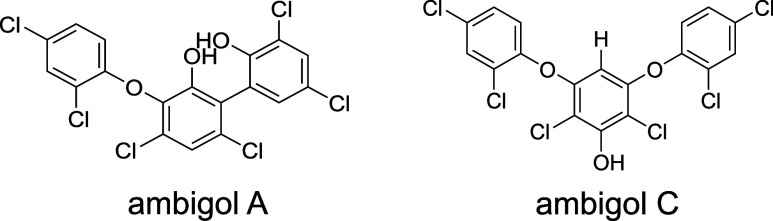
Structures of ambigols A and C.

Ambigols are known to possess a broad bioactivity spectrum. Ambigol A, B, and C were found to be antibacterial and antifungal.^14,15^ Ambigol A and B were in addition shown to be antiviral, cytotoxic, molluscicidal, and inhibitors of the enzyme cyclooxygenase.^14^ Ambigol A and C were demonstrated to be antiplasmodial and trypanocidal, ambigol A also antialgal.^15^ In these studies, ambigol A was in general biologically more active than the other ambigol congeners.^14,15^

In previous work, we demonstrated that the ambigols, especially ambigol C, increase the production of the metabolite prodigiosin in *Serratia* sp. 39006.^16^ Prodigiosin is a compound with potential applications in the clinical, environmental, and nutritional sectors because of its biological activities.^17^ Changes in the transcriptome of ambigol C-treated *Serratia* sp. 39006 were found for genes related to metabolic pathways, oxidative phosphorylation, amino acid and sugar transport, chemotaxis, and ribosomal function.^16^ Molecules structurally closely related to ambigol C, like pentachlorophenol, also influence the chemotaxis and amino acid transport in *Bacillus subtilis* ^18–20^ inhibit the proton-sugar-transport, and influence the RNA, protein, and ribosome biosynthesis of *Saccharomyces* species.^21–23^ Like aetokthonotoxin, chlorophenols are known uncouplers of oxidative phosphorylation.^24^

Given the structural similarities of the compounds mentioned above, we hypothesized that the ambigols act as uncouplers of oxidative phosphorylation. Due to our previous work on prodigiosin synthesis mainly emphasizing on ambigol C, we chose to test this hypothesis for ambigol C in *Escherichia coli* and *Serratia* sp. 39006 (meanwhile renamed to *Prodigiosinella confusarubida* ^25^ or *Prodigiosinella aquatilis* ^26^). In the following, we will refer to it as *P. aquatilis*. We further tested our hypothesis for ambigol C and the highly bioactive ambigol A in eukaryotic HeLa cells. Eventually, we demonstrate that ambigols A and C indeed act as uncouplers of oxidative phosphorylation.

## Results and Discussion

### Ambigol C Affects the Membrane Potential in Gram-Negative Bacteria

In our previous work, we tested the effects of ambigol C on the transcriptome and prodigiosin production of the Gram-negative bacterial strain *P. aquatilis* (*Serratia sp.* 39006).^16^ Therefore, we chose this strain together with the well-established Gram-negative bacterial strain *Escherichia coli* as model organisms for our study on the mode of action of ambigol C.

After ascertaining the minimal inhibitory concentration of ambigol C for both strains (>100 μM), we kinetically monitored the membrane potential, ΔΨ, in *P. aquatilis* and *E. coli* upon acute treatment with ambigol C during 2 h. In the first experiment, we assessed ΔΨ in energized cells. As the direction of the membrane polarization can be a matter of change depending on media composition and nutrient supply,^2^ we repeated the experiment with de-energized cells kept in phosphate-buffered saline (PBS) instead of lysogeny broth (LB).

We observed hyperpolarization of the membrane of energized *P. aquatilis* in a concentration-dependent manner directly after treatment (Figure 2A). *P. aquatilis* recovered the membrane potential quickly when treated with lower to medium concentrations of ambigol C. Higher concentrations, however, led to a prolonged increase of membrane polarization, peaking 15 min (50 μM ambigol C) or 45 min (100 μM ambigol C) after treatment, while the known uncoupler 2-[[4-(trifluoromethoxy)­phenyl]­hydrazinylidene]­propane-di-nitrile (FCCP) had no effect. In de-energized *P. aquatilis* however, treatment with the same FCCP concentration resulted in a marked depression of the membrane potential, indicating enhanced toxicity, and a higher susceptibility of these cells to FCCP. Different ambigol C concentrations had contrasting effects on the membrane potential in *P. aquatilis*. The lower concentrations evoked no effect, while 15 and 50 μM ambigol C showed a trend in hyperpolarizing the membrane, being statistically significant after 75 min of treatment with 50 μM ambigol C. At the highest concentration tested, 100 μM, the membrane hypopolarized directly after treatment and did not recover during the time of the experiment (Figure 2B).

**Figure 2:**
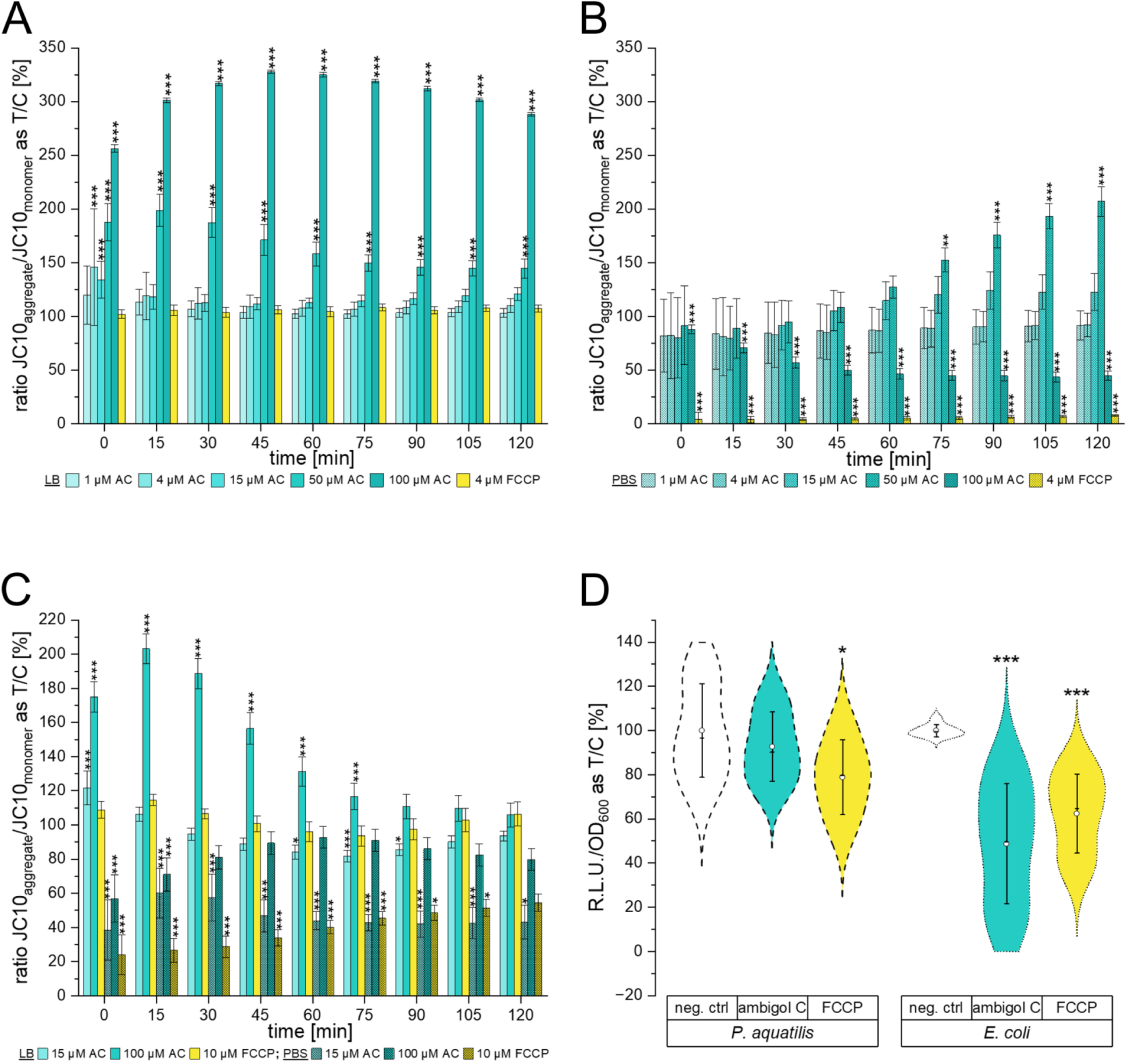
(A) Membrane potential of *P. aquatilis* assessed in LB-medium respective to control. (B) Membrane potential of *P. aquatilis* assessed in PBS respective to control. (C) Membrane potential of *E. coli* assessed in LB-medium (open bars) or PBS (hatched bars) respective to control. (D) ATP level of *P. aquatilis* (dashed plots on the left) or *E. coli* (dotted plots on the right) normalized to the optical density, respective to the negative control. Mean is depicted as a white dot, median as a black line. (A) – (D) AC, ambigol C; neg. ctrl, negative control (solvent); *p < 0.05, ** *p* < 0.001, *** *p* < 0.0001 determined by ANOVA (A-C) or Student’s *t* test (D) of three independent biological replicates; R.L.U. relative luminescence units; T/C treatment over control; Whiskers indicate 1× standard deviation.

In *E. coli*, we subsequently tested a reduced set of ambigol C concentrations. In energized *E. coli* cells, 100 μM ambigol C also led to a hyperpolarized membrane. The peak was however already reached after 15 min incubation time and then decreased more rapidly compared to the effect of the same ambigol C concentration on *P. aquatilis*. 15 μM of ambigol C also evoked a brief increase of membrane polarization at the beginning of the experiment in *E. coli*, while FCCP, again, showed no effect (Figure 2C). *E. coli* maintained in PBS and treated with 15 μM ambigol C or FCCP showed a hypopolarization of the membrane throughout the whole experiment. This difference was statistically significant compared to the control, as it was for 100 μM ambigol C for the first 15 min of the experiment. Then, *E. coli* quickly recovered the membrane potential under influence of the highest concentration tested. Bacterial electrophysiology comprises the homeostatic control of uniquely combined, nonlinearly coupled variables.^27^ Hence, a nonlinear response to electrophysiologically interfering stimuli might result from the homeostatic control of each of these variables. With these assays we demonstrate that ambigol C disturbs the membrane potential in both bacterial strains in a comparable manner, and that *P. aquatilis* and *E. coli* strive to restore it.

The ability of *E. coli* to adjust to altered electrochemical conditions is known. Skulachev has reviewed the research demonstrating the ability of *E. coli* to switch from proton-driven ATP-synthesis to sodium-driven ATP synthesis in the presence of an uncoupler or in an alkaline environment. This ability to rely on Na^+^ instead of H^+^ for oxidative phosphorylation renders *E. coli* and other bacterial strains able to cope with the impact of protonophores.^28^

The activity of a primary respiratory sodium pump was further considered responsible for the accumulation of proline in a protonophore-resistant *E. coli* strain.^29,30^ l-Proline is a known precursor in prodigiosin biosynthesis in *P. aquatilis*.^31^ Interestingly, Chilczuk et al. discovered that the prodigiosin biosynthesis in in *P. aquatilis* is increased after treatment with ambigol C. They detected an incorporation of 5-oxo-l-proline in prodigiosin, and attributed this to reduced gene transcript levels of an ATP-dependent 5-oxoprolinase.^16^ In this regard, it might be worth to monitor the abundance of l-proline in *P. aquatilis* after treatment with ambigol C in future work, as it might also contribute to the total augmentation of prodigiosin biosynthesis.

The membrane potential is intertwined with ATP synthesis in the process of oxidative phosphorylation.^32^ In the light of the aforementioned sodium-based compensation of protonophore-induced interferences with oxidative phosphorylation, the assessment of ATP as a complementary end point would mean capturing a rather transient state. To maximize the chance to detect any changes in total ATP content of *P. aquatilis* and *E. coli*, we chose to test the ambigol C concentration and incubation period that resulted in the most pronounced effect on the respective bacterial membrane potential.

Although the membrane potential was similar to the control when energized *P. aquatilis* and *E. coli* were treated with FCCP, the total amount of ATP was statistically significantly lower compared to the control (Figure 2D). This suggests that the membrane potential could only be preserved under some expense of ATP when bacteria were stimulated with FCCP.

In *P. aquatilis*, ATP content after stimulation with 100 μM ambigol C did not differ from the control. *E. coli* on the contrary showed a statistically significant reduction of ATP amount compared to the control. ATP- and proton motive force-dependent efflux pumps are often used in bacteria to extrude toxic substances, like protonophores, thereby mitigating their impact on the cells.^33,34^ A protonophore similar to FCCP, carbonyl cyanide-*m*-chlorophenyl hydrazone (CCCP), was shown to heterogeneously dissipate the proton motive force (pmf) in a population of *E. coli* due to active efflux of the compound.^34^ Active efflux could possibly contribute to the heterogeneous distribution pattern of ATP content depicted in Figure 2D as it might determine the availability of FCCP and ambigol C in the *E. coli* population, as well. Also, because both *P. aquatilis* and *E. coli* are facultatively anaerobic, they can switch to ATP production pathways that do not rely on the respiratory chain.

In contrast to *E. coli*, *P. aquatilis* is pigmented. Its red pigment, prodigiosin, was shown to be associated with the cellular ATP levels in *Serratia marcescens* during different growth stages.^35^ The complex regulation of prodigiosin synthesis in *P. aquatilis* was to some extent linked to energy metabolism and cellular oxygen levels.^36,37^ In studies on liposomes, it was demonstrated that prodigiosin is an ionophore, acting as an H^+^/Cl^¯^ symporter and/or anion exchanger, namely Cl^¯^/NO_3_^¯^ antiporter.^38,39^ Ambigol C stimulates the prodigiosin production in *P. aquatilis*: It affects the regulation of metabolic pathways, oxidative phosphorylation, nitrogen metabolism, and flagellar assembly.^16^ We show here that ambigol C interferes with the bacterial membrane potential. Besides energy metabolism in terms of transport and oxidative phosphorylation, the membrane potential is involved in bacterial motiliy,^40,41^ environmental sensing,^42–44^ and electrical communication.^45,46^ Future studies could therefore focus on the physiological role of prodigiosin for *P. aquatilis* in coping with uncoupler-induced bioenergetic challenges.

The results so far did not unequivocally demonstrate uncoupling of oxidative phosphorylation in prokaryotes, since the membrane potential is only part of the proton motive force that drives oxidative phosphorylation. However, the results provide strong evidence, as it is known that compounds affecting the membrane potential can cause uncoupling.^1^ Regarding electrophysiological tools and physicochemical models, more paradigms exist for eukaryotic than for prokaryotic cells.^27,44^ Uncouplers are well characterized by their activity on mitochondria in eukaryotic cells. Thus, to test our hypothesis in more depth, we decided to assess ambigol-induced uncoupling of oxidative phosphorylation in HeLa cells.

### Ambigols A and C Uncouple Oxidative Phosphorylation in Eukaryotic Cells

The mitochondrial membrane potential (ΔΨ_m_), together with the proton gradient (ΔpH), composes the mitochondrial proton motive force (pmf). This force is sustained through the respiratory chain, and consumed mainly by the F_o_F_1_-ATPase during ATP synthesis. Uncouplers disturb this interplay by dissipating the pmf.^32^ To do so, protonophoric uncouplers need a functional group that is able to release and capture protons. Ambigol C has one such hydroxyl group, whereas the structurally related ambigol A has two (Figure 1). According to our calculations, the respective p*K*_a_ values are 7.2 for ambigol C, and 6.7 for ambigol A (Table S1), which lies in the optimum range for weakly acidic uncouplers.^47^

As a response to uncoupler induced pmf-dissipation, cells adjust their respiratory chain activity to restore ΔΨ_m_ and ΔpH. Thereby, more oxygen is consumed.^48^ We used a Seahorse analyzer to assess the oxygen consumption rate (OCR) in HeLa cells. As we assumed that the ambigols A and C uncouple the oxidative phosphorylation, we first inhibited the mitochondrial F_o_F_1_-ATPase with oligomycin to prevent its reverse ATPase activity that could mask slight uncoupling effects. Subsequently, we treated the cells with ambigols A and C, or FCCP as a positive control. Any rise in OCR upon these treatments would now be attributable to disturbances of the pmf followed by compensatory respiratory chain activity. Indeed, we observed a concentration dependent increase of OCR after addition of ambigol C (Figure 3A). For ambigol A, 6 μM evoked a pronounced escalation of OCR, while 25 μM ambigol A caused only a slight rise. This is typical for uncouplers, as they have a bell-shaped effect curve on OCR.^49^ After inhibiting the total respiratory chain activity with rotenone and antimycin A, the OCR decreased accordingly, showing no difference in extra-mitochondrial oxygen consumption between the treatments. With these results, we demonstrate that ambigol A and C are uncouplers of oxidative phosphorylation in HeLa cells—as suspected due to the close structural similarity of the ambigols to the PBDEs, which have the same activity. Furthermore, our results indicate a stronger uncoupling activity of ambigol A compared to ambigol C.

**Figure 3:**
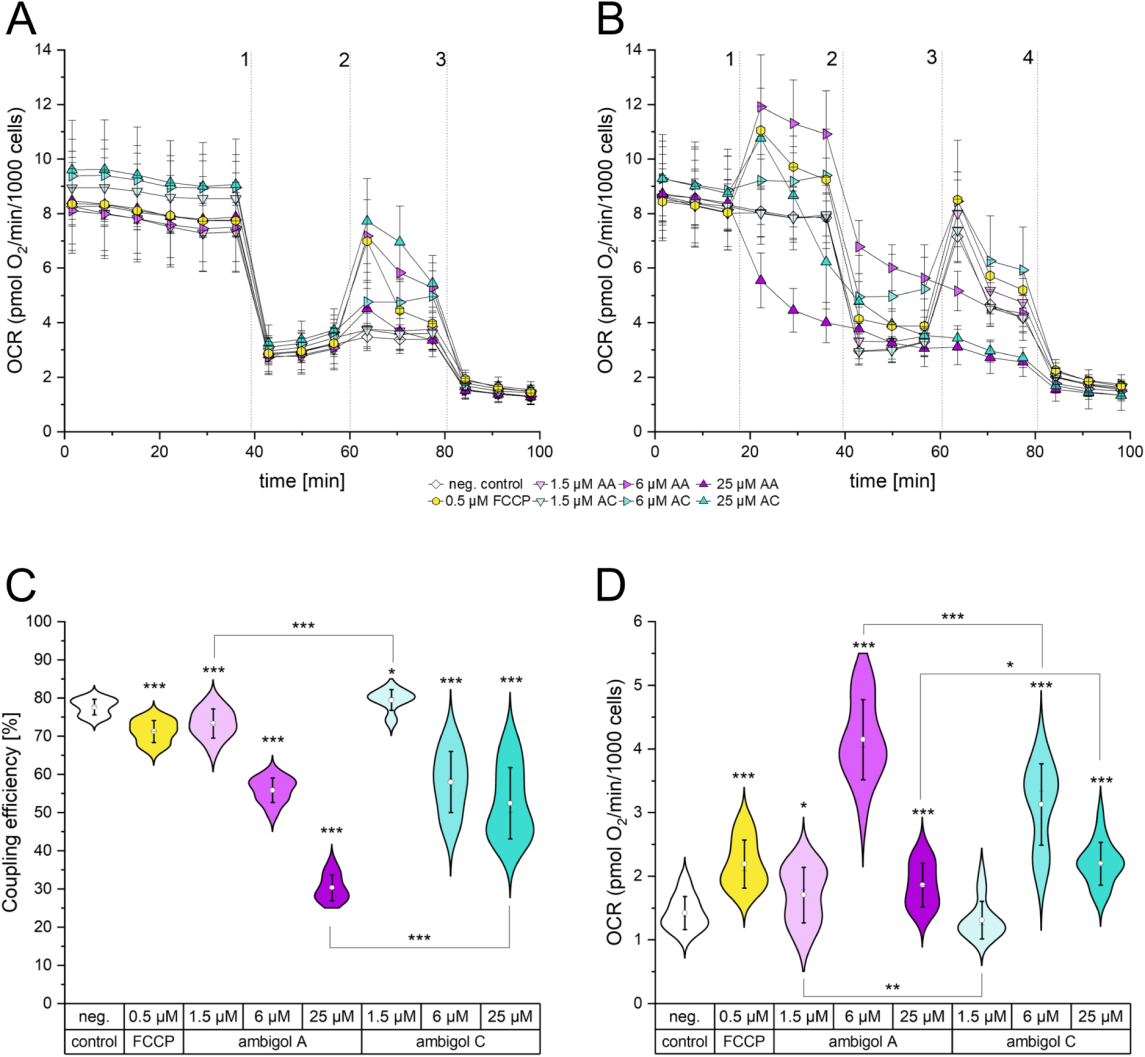
(A) Oxygen consumption rate of HeLa cells after acute stimulation with ambigol A, ambigol C, or FCCP subsequent to F_o_F_1_-ATPase inhibition. Addition of 1: oligomycin, 2: ambigol or FCCP, 3: rotenone/antimycin A. (B) Oxygen consumption rate of HeLa cells after acute stimulation with ambigol A, ambigol C, or FCCP. Addition of 1: ambigol or FCCP, 2: oligomycin, 3: FCCP, 4: rotenone/antimycin A. (C) Coupling efficiency of HeLa cells in response to stimulation with ambigol A, ambigol C, or FCCP. (D) Proton leak of HeLa cells in response to stimulation with ambigol A, ambigol C, or FCCP. (A) – (D) AA, ambigol A; AC, ambigol C; neg. control, negative control (solvent); OCR, oxygen consumption rate; Whiskers indicate 1× standard deviation. (C) – (D) Mean is depicted as a white dot, median as a black line; * *p* < 0.05, ** *p* < 0.01, *** *p* < 0.001 determined by Student’s *t* test of three independent biological replicates.

The uncoupler-induced maximal capacity of the mitochondrial electron transport system is often underestimated in the presence of oligomycin.^50^ In the next step, we thus tested the effects of ambigols A and C on noninhibited oxidative phosphorylation, as it would be the case in nature. We changed the experimental setting to a primary addition of three concentrations of ambigol A and C, respectively (Figure 3B). This was then followed by additions of oligomycin, FCCP and rotenone/antimycin A to assess the coupling efficiency, proton leak, and respiratory chain activity. By doing so, we observed OCR trajectories that were similar to the control for 1.5 μM ambigol A and C, and 6 μM ambigol C. Six μM ambigol A led to a steep increase of OCR, as did the positive control FCCP. After a short peak, OCR declined rapidly after treatment with 25 μM of ambigol C. The same concentration of ambigol A led directly to a decreasing OCR after injection. In both cases the cells did not respond to subsequent stimulation with oligomycin, or FCCP. Also, cells treated with 6 μM ambigol A did not respond to succeeding stimulation with FCCP. This finding indicates that these treatment concentrations have already exhausted the cellular capacities to respond to further respiratory chain stimulation. Since all OCRs accumulated on the same level after injection of rotenone/antimycin A, we can conclude that the detected changes in OCRs are attributable to changes in mitochondrial OCRs. The same effects were observed for ambigol A and C, albeit the concentrations at which these effects occurred were lower for ambigol A than for ambigol C throughout the experiment. The difference in effect intensity between ambigol A and C is additionally reflected in the impairment of mitochondrial coupling efficiency, and the effect on proton leak, as shown in Figure 3C and 3D, respectively. Proton leak was increased by every treatment concentration apart from 1.5 μM ambigol C. 6 μM ambigol A and C led to the highest proton leak. The comparatively lowered proton leak induced by 25 μM ambigol A and C can be explained by the rather similar OCRs after oligomycin and FCCP stimulation. Thus, in this case, proton-leak-linked and uncoupler-stimulated respiration seem matched, indicating changes in ΔΨ_m_.^48^

We next assessed the influence of ambigols A and C on ΔΨ_m_ in HeLa cells in a time-kinetic experiment. We observed that every treatment with ambigol A or C eventually resulted in ΔΨ_m_ dissipation. The data distribution depicted in Figure 4A shows that a higher treatment concentration of either ambigol A or C is more efficiently reducing ΔΨ_m_ in a shorter period of time than lower concentrations. This experiment demonstrates that ambigols A and C time- and concentration-dependently affect the ΔΨ_m_.

**Figure 4:**
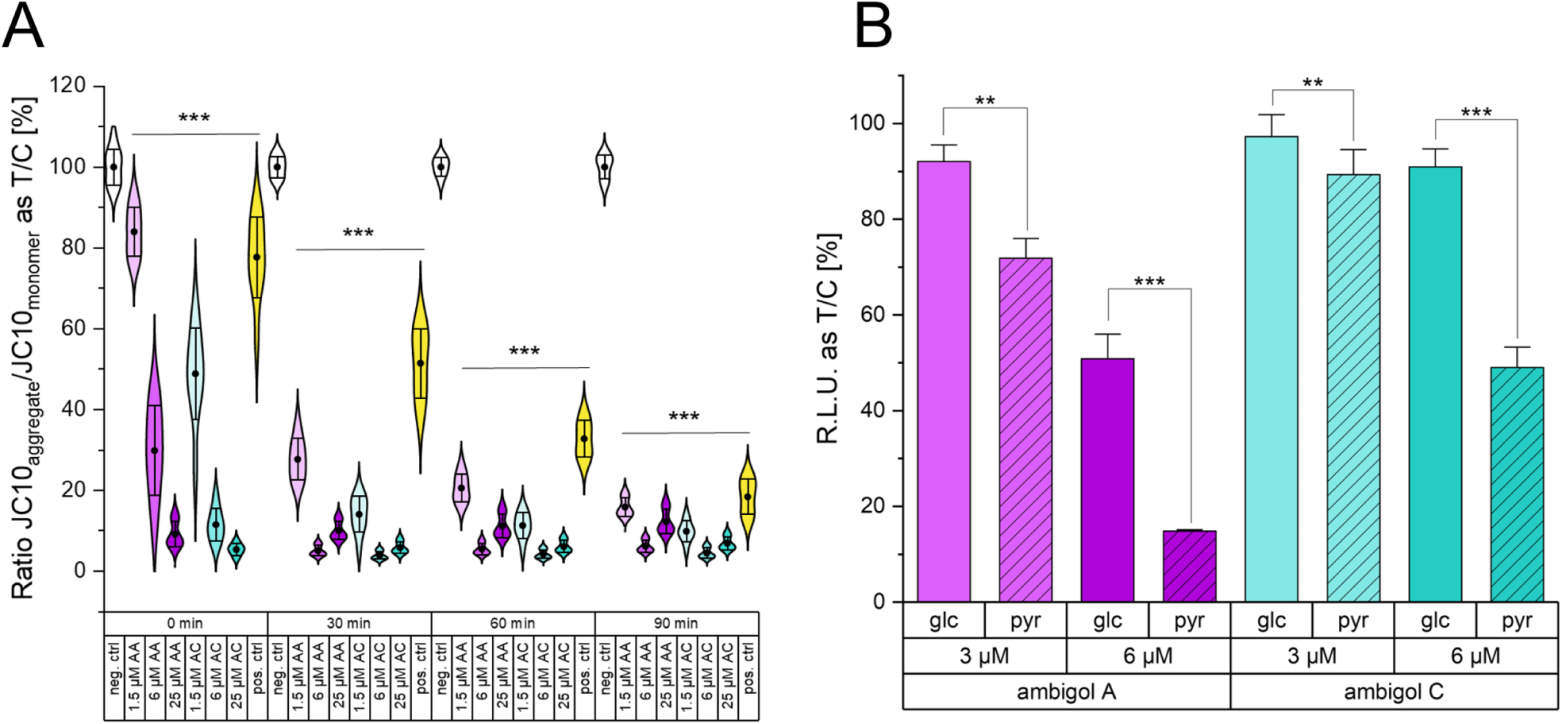
(A) Mitochondrial membrane potential of HeLa cells respective to the negative control. AA, ambigol A; AC, ambigol C; neg. ctrl, negative control (solvent); pos. ctrl, positive control (FCCP). Mean is depicted as a black dot. (B) ATP level of HeLa cells after treatment with ambigol A or ambigol C in glucose (glc) or pyruvate (pyr) containing media respective to the control. R.L.U. relative luminescence units. (A) – (B) ** *p* < 0.01, *** *p* < 0.001 determined by Mann–Whitney (A) or Student’s *t* test (B) of three independent biological replicates; T/C treatment over control; Whiskers indicate 1× standard deviation.

Having determined the impact of ambigols A and C on mitochondria, we were eventually interested in the bioenergetic consequences for the HeLa cell. Hence, we assessed the ATP level of metabolically manipulated HeLa cells by the provision of different nutrient supplies before treatment with ambigol A or C. To this aim, we incubated HeLa cells in pyruvate containing medium, to force mitochondrial ATP synthesis, or in high-glucose containing medium that stimulates anaerobic ATP generation.^51^ We compared the toxicity of ambigol A and C on both cell cultivates, and found a statistically significant enhanced vulnerability of the cells that were constrained to oxidative phosphorylation (Figure 4B). This result demonstrates that ambigol A and C are affecting mitochondrial activity *in vitro*. It is furthermore in line with our finding that ambigol A and ambigol C are both uncouplers of oxidative phosphorylation.

### Ambigols Uncoupling Oxidative Phosphorylation Is Compliant with Existing Data

In 2023, Milzarek et al. made a first attempt to elucidate the mode of action of ambigols by examining simplified synthetic ambigol derivatives in a biosensor assay utilizing *Bacillus subtilis*. They detected a stress response related to cell membrane integrity, and a negative effect on DNA repair. The authors discussed the similarity of these results regarding the mode of action of the structurally closely related triclosan, and suggested a potentially bifunctional mode of action of the ambigols.^52^ Triclosan, however, is also known as an uncoupler of oxidative phosphorylation and as a protonophore, affecting the membrane potential of *B. subtilis*.^53–55^ In more detail, Milzarek et al. observed the induction of a promoter regulating an operon that determines the cell membrane fluidity, indicating its decrease after treatment of *B. subtilis* with the ambigol derivatives. Interestingly, treatment of *B. subtilis* with CCCP resulted in the downregulation of a gene that indicated the same outcome, hence lowered membrane fluidity.^56^ CCCP, in turn, also uncouples oxidative phosphorylation as weakly acidic uncoupler.^1,57^

As the ambigols are biosynthesized from 2,4-dichlorophenol building blocks,^13^ each ambigol still possesses a dissociable proton which is essential for protonophore activity.^11,58^ Indeed, Milzarek et al. confirmed that methylation of the free hydroxy groups rendered the respective ambigol derivatives mostly inactive in their assays for antibacterial and antifungal activity. Surprisingly, two of their methylated derivatives retained some bioactivity, albeit it was much reduced compared to the respective unmethylated derivatives. Interestingly, in contrast to antimicrobial activity, cytotoxicity of the methylated derivatives and unmethylated derivatives was comparable, which could indicate that either protonophore activity might not be the sole mechanism behind the uncoupling,^49,52^ or that the xenobiotics metabolism in the eukaryotic cells demethylates the derivatives to the respective active metabolites. Given their very similar calculated p*K*_a_ and log *P* values (Table S1), it is also surprising that 3,5-dichloro-2-(3,5-dichlorophenoxy)­phenol was found to be biologically inactive, while the structurally very closely related 2,4-dichloro-6-(3,5-dichlorophenoxy)­phenol was found to be active in the assays.^52^ We could not deduce any strong relationship between the bioactivity of the reported ambigol derivatives and their calculated p*K*_a_ values. Most likely, other parameters like membrane composition, membrane permeability or efflux pump transportability might also influence the activity of the ambigol derivatives besides their protonophore activity, which was also suggested by the reported differential toxicity for *C. elegans*.^52^ However, we observed that a p*K*_a_ value >8 might be advantageous for the antifungal activity determined by Milzarek et al.^52^

Because of the ubiquity of oxidative phosphorylation in energy conversion across the kingdoms of life, the successful development of ambigol-based compounds, in particular as antibiotics, will require profound investigations into species-specific susceptibility with emphasis on appropriate safety margins.

## Conclusion

We discovered that ambigol C disturbs the membrane potential in *E. coli* and *P. aquatilis*. It remains an open question how this impact mechanistically influences the bacterial cell signaling leading to increased prodigiosin production in *P. aquatilis*. Given the importance of the membrane potential for electrical communication^44–46^ and the regulation of prodigiosin production by quorum-sensing^59^ further research in this direction seems a worthwhile endeavor.

Moreover, we demonstrate that ambigol A and ambigol C dissipate the mitochondrial membrane potential in HeLa cells, affecting mitochondrial ATP synthesis. We showed that ambigol A and C are typical uncouplers of oxidative phosphorylation. Since uncoupling is an unspecific process, this mode of action might underlie the broad bioactivity spectrum of the ambigols. Further development of ambigols for applications in any field should take these findings into account.

## Methods

Ambigol A, ambigol C, and FCCP were dissolved in dimethyl sulfoxide (DMSO) (AppliChem, Germany), and diluted in the respective media used in the individual experiments. The respective solvent control was always carried along during the course of each experiment. Every experiment was conducted in three independent biological replicates consisting of three technical replicates, respectively.

### Microbiology

#### Cultivation

*Escherichia coli* DH5α (DSM 6897) and *Prodigiosinella aquatilis* (ATCC 39006) were cultivated on LB-agar plates (Sigma-Aldrich, USA) at 37 °C and 30 °C, respectively.

#### Minimal Inhibitory Concentration

The minimal inhibitory concentrations (MIC) of ambigol C and FCCP were determined according to the guidelines of the Clinical and Laboratory Standards Institute (CLSI) using the broth microdilution method.^60^ Briefly, the compounds were dissolved in DMSO and diluted in LB-medium (Sigma-Aldrich, USA) to various concentrations of 0.01–60 μM (FCCP) or 120 μM (ambigol C) in a clear 96-well plate (Greiner Bio-One, Germany). Colonies were picked, suspended to an optical density (OD_600_) of 0.08–0.10, and diluted 20-fold to inoculate a total of 5 × 10^4^ CFU/well. After 20 h of incubation with the compounds at 37 °C (*E. coli*) or 30 °C (*P. aquatilis*), the bacterial growth was determined by measuring the OD at 620 nm using a Tecan Infinite 200 Pro M Plex plate reader (Tecan Group, Switzerland). Growth was defined by an OD_600_ of 0.02 higher than that of the uninoculated control.

#### Bacterial Membrane Potential

The assessment of bacterial membrane potential was based on the procedure described by Cléach et al.^61^ Bacteria in the stationary phase were inoculated in fresh medium and grown on an orbital shaker with 120 rpm at 37 °C (*E. coli*) or 30 °C (*P. aquatilis*) to reach the exponential phase. Bacteria were harvested, and the OD_600_ was adjusted to 0.3. According to the experimental setting, the bacteria suspension was centrifuged and resuspended in PBS or LB-medium. The suspension was then plated into a black 96-well plate with transparent bottom, and incubated with JC-10 from the JC-10 Membrane Potential Assay Kit for microplates (no. AB112134, Abcam, Cambridge, UK) for 1 h at the respective temperature. The kit was applied following the manufacturer’s instructions. Afterward, bacteria were acutely stimulated with our compounds of interest and the membrane potential was instantaneously monitored for 2 h every 15 min by measuring the fluorescence of JC-10 with a Tecan Infinite 200 Pro M Plex plate reader at excitation/emission 490/525 nm and 540/590 nm.

#### Bacterial ATP Level

Bacteria from the exponential phase (see method on bacterial membrane potential) were treated for 15 min (*E. coli*) or 45 min (*P. aquatilis*) with ambigol C or FCCP diluted in LB-medium in a black 96-well plate with transparent bottom. Shortly before the end of the incubation period, the OD_600_ for each well was determined using a Tecan Infinite 200 Pro M Plex plate reader. The BacTiter-Glo Kit from Promega (Promega, USA) was then applied according to the manufacturer’s instructions to assess ATP levels. In brief, the BacTiter-Glo reagent was added in equivalent volumes to the wells. The plate was then orbitally shaken for 30 s, and luminescence was recorded after 5 min with the same plate reader. The relative luminescence units for each well were adjusted to the background and normalized to the respective OD_600_ value.

### Cell Biology

#### Cultivation

Human cervix carcinoma HeLa cells (provided by Prof. Junker, Martin Luther University, Halle-Wittenberg, Germany), were maintained at 37 °C in a humidified atmosphere with 5% CO_2_. Cells were passaged after reaching 80–90% confluence. HeLa cells were cultivated in Dulbecco’s Modified Eagle Medium (DMEM) with low glucose (Carl Roth, Germany) supplemented with 10% (v/v) fetal bovine serum (Sigma-Aldrich, USA) and 2 mM glutamine (Carl Roth, Germany).

#### Oxygen Consumption and Proton Efflux Rate Measurements

A Seahorse XFe96 Analyzer (Agilent, USA) was used to assess the cellular oxygen consumption rate (OCR) and proton efflux rate (PER). 2 × 10^4^ HeLa cells were seeded per well in Seahorse XF cell culture microplates (Agilent, USA) and allowed to attach for 24 h. On the day of the experiment, medium was replaced with Seahorse XF DMEM, pH 7.4, supplemented with 10 mM glucose, 2 mM glutamine, and 1 μM pyruvate (all Agilent, USA).

The Seahorse XF Cell Mito Stress Kit (Agilent, USA) was prepared according to the manufacturer’s instructions. The hydrated sensor cartridge was equipped according to the experimental requirements with various concentrations of ambigol A or C or solvent control and the kit components as follows: 2 μM oligomycin, 0.5 μM FCCP, and 0.5 μM rotenone + antimycin A (RAA). These concentrations and seeding densities were determined as optimal in preliminary experiments.^11^ After 1 h of incubation at 37 °C without CO_2_, the plate was placed into the Seahorse XFe96 Analyzer to assess OCR and PER at 37 °C. Each measurement consisted of 3 min mixing and 3 min measuring, and was repeated three times. OCR and PER were normalized to the cell mass per well, that was determined in a subsequent SRB assay as outlined below. The software Wave Pro v. 10.2.1.4 (Agilent, USA) was used for data analysis. Coupling efficiency and proton leak were calculated according to the Agilent user guide RA.4773611111.16.

#### Sulforhodamine B Assay

The sulforhodamine B (SRB) colorimetric assay was performed as previously described with slight modifications.^62^ In brief, immediately adjacent to the Seahorse assay, HeLa cells were fixed with cold 10% (w/v) trichloroacetic acid, and kept at 4 °C for 1 h. Afterward, cells were carefully rinsed four times with slow-running tap water, blow dried, and stored at RT. On the day of the experiment, 0.057% (w/v) SRB solution (Sigma-Aldrich, USA) in 1% (v/v) acetic acid was added to each well. After 30 min of incubation time at RT, the cells were quickly washed four times with 1% (v/v) acetic acid, and blow dried. 10 mM Tris base solution (pH 10.5) was added to the completely dry wells. The plate was placed in a Tecan Infinite 200 Pro M Plex plate reader, orbitally shaken for 300 s, and absorbance was recorded at 510 nm.

#### Mitochondrial Membrane Potential

HeLa cells were seeded in a black 96-well plate with a flat, clear bottom and allowed to attach overnight. The mitochondrial membrane potential was monitored kinetically, measuring instantly after addition of ambigol A, ambigol C, and FCCP to the wells, and subsequently every 30 min for a total of 90 min. The JC-10 Mitochondrial Membrane Potential Assay Kit for microplates (no. AB112134, Abcam, Cambridge, UK) was applied following the manufacturer’s instructions. Fluorescence of JC-10 was recorded with a Tecan Infinite 200 Pro M Plex plate reader at excitation/emission 490/525 nm and 540/590 nm.

#### Cellular ATP Level

The total ATP level was determined in a luminescence-based assay using the CellTiter-Glo Kit from Promega (Promega, USA) according to the manufacturer’s instructions. Briefly, HeLa cells were seeded into opaque-walled 96-well plates, and cultivated in two different media: DMEM-glucose or DMEM-pyruvate. DMEM-glucose consisted of DMEM high glucose (25 mM) + 10% FBS + penicillin (100 IU/mL) + streptomycin (100 mg/L) + 2 mM glutamine + 25 mM HEPES), and DMEM-pyruvate was composed of DMEM no glucose + 5 mM pyruvate + 10% FBS + penicillin (100 IU/mL) + streptomycin (100 mg/L) + 2 mM glutamine + 25 mM HEPES). The next day, cells were incubated with different concentrations of ambigol A and C for 24 h in the respective media. After the incubation time, the cells were allowed to reach RT for 30 min. Then, the CellTiter-Glo reagent was added in equivalent volumes to the wells, and the plate was orbitally shaken for 2 min. After 10 min settling time, luminescence was recorded using a Tecan Infinite 200 Pro M Plex plate reader.

#### Statistics

OriginPro 2025 software v. 10.2.0.196 (OriginLab Corporation, USA) was used for statistical analysis that was conducted with the data obtained from each technical replicate. Data distribution was assessed with the Lillie-Force test for normality. Outliers were identified applying Grubbs test if necessary. Parametric data was assessed using a two-sided Student’s *t* test, while nonparametric data was assessed using a Mann–Whitney test. For the data on bacterial membrane potential, a two-way ANOVA with a Tukey post hoc test was performed.

#### Calculation of p*K*_a_ and log *P*

MOE 2022.02 (Molecular Operating Environment; Chemical Computing Group ULC, Montreal, QC, Canada) with the “Calculate Descriptors” functionality was used to calculate the 2D descriptors log *P*(o/w) and h_pKa.^63^

## Supplementary Material



## Data Availability

Primary data for the oxygen consumption rate measurements, membrane potential assays, and ATP quantifications have been uploaded to figshare (10.6084/m9.figshare.31430134).

## Notes

Calculated p*K*_a_ and log *P* values, and structures of ambigol derivatives (PDF)
