# Supplementary material for: Ambigols Uncouple Oxidative Phosphorylation In Vitro
Source: ACS Chem Biol. 2026 Apr 2;21(4):689–97. doi: 10.1021/acschembio.5c00952 (PMC13097137; doi:10.1021/acschembio.5c00952)
Supplement: Supplementary file 1 [file cb5c00952_si_001.pdf]

Supporting Information

Ambigols Uncouple Oxidative Phosphorylation In Vitro

Valerie I. C. Rebhahn[1], Clemens A. Wolf[2], Timo H. J. Niedermeyer[1,*]

[1] Institute of Pharmacy, Pharmaceutical Biology, Freie Universität Berlin, Berlin 14195, Germany

[2] Department of Biomedicine, University of Bergen, Bergen 5020, Norway

Table S1. Calculated pK_a and logP values for ambigol derivatives reported by Milzarek *et al.*[1] and their structures. a: R = H, b: R = CH_3

Ambigol	Compound[1]	pKa	logP(o/w)	Compound[1]	pKa	logP(o/w)
A	1	6,7	> 5.5			
B	2	6,7	> 5.5			
C	3	7,2	> 5.5			
D	4	6,8	> 5.5			
E	5	6,7	> 5.5			
	6	7,1	> 5.5			
	7	7,2	> 5.5			
	8a	7,6	> 5.5	8b	> 14	> 5.5
	9a	7,5	> 5.5	9b	> 14	> 5.5
	10a	8,3	> 5.5	10b	> 14	> 5.5
	11a	7,6	> 5.5	11b	> 14	> 5.5
	12a	7,5	> 5.5	12b	> 14	> 5.5
	13a	8,3	> 5.5	13b	> 14	> 5.5
	14a	7,2	> 5.5	14b	> 14	> 5.5
	15a	7,3	> 5.5	15b	> 14	> 5.5
	16a	7,4	> 5.5	16b	> 14	> 5.5

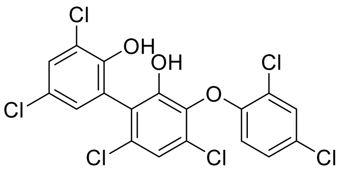
ambigol A (**1**)

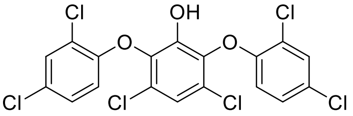
ambigol B (**2**)

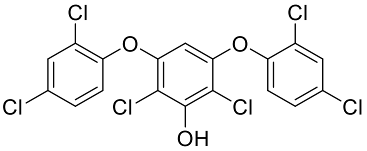
ambigol C (**3**)

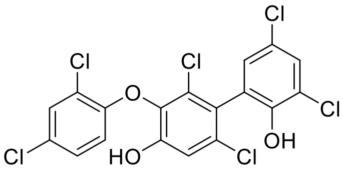
ambigol D (**4**)

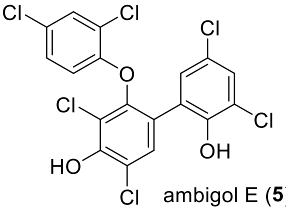
ambigol E (**5**)

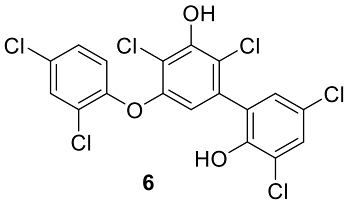
6

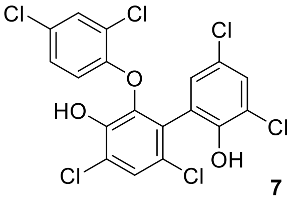
7

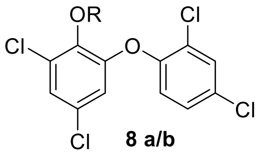
8 a/b

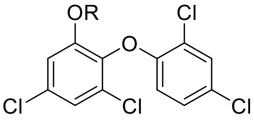
9 a/b

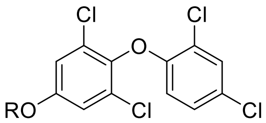
10 a/b

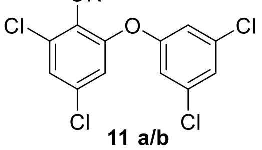
11 a/b

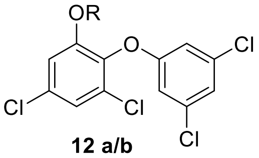
12 a/b

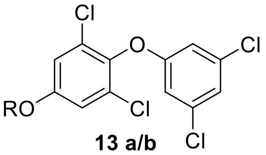
13 a/b

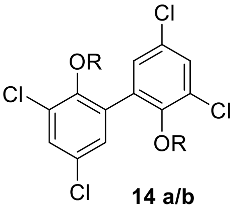
14 a/b

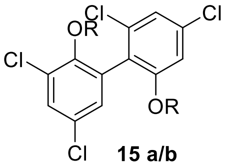
15 a/b

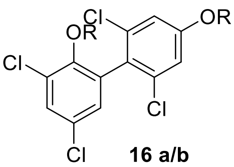
16 a/b

---

1 Milzarek, T. *et al*. ACS Infect. Dis. 2023, 9, 1941–1948.
